# Conditional ablation of the prorenin receptor in nephron progenitor cells results in developmental programming of hypertension

**DOI:** 10.14814/phy2.13644

**Published:** 2018-04-02

**Authors:** Renfang Song, Laura Kidd, Adam Janssen, Ihor V. Yosypiv

**Affiliations:** ^1^ Department of Pediatrics Tulane University School of Medicine New Orleans Los Angeles; ^2^ Department of Pathology Tulane University School of Medicine New Orleans Los Angeles

**Keywords:** Developmental programming, hypertension, kidney development, nephrogenesis, nephron progenitor cells, prorenin receptor

## Abstract

Nephron induction during kidney development is driven by reciprocal interactions between progenitor cells (NPCs) of the cap mesenchyme (CM) and the ureteric bud (UB). The prorenin receptor (PRR) is a receptor for renin and prorenin, and an accessory subunit of the vacuolar proton pump V‐ATPase. Previously, we demonstrated that conditional ablation of the *PRR* in Six2^+^ NPCs in mice (*Six2* ^PRR^ ^−/−^) causes early neonatal death. Here, we identified genes that are regulated by PRR in Six2^+^ NPCs FACS‐isolated from *Six2* ^PRR^ ^−/−^ and control kidneys on embryonic day E15.5 using whole‐genome expression analysis. Seven genes with expression in CM cells previously shown to direct kidney development, including *Notch1*,* β*‐catenin, *Lef1, Lhx1, Jag1, and p53,* were downregulated. The functional groups within the downregulated gene set included genes involved in embryonic and cellular development, renal regeneration, cellular assembly and organization, cell morphology, death and survival. Double‐transgenic *Six2* ^PRR^ ^−/−^/*BatGal* ^+^ mice, a reporter strain for *β*‐catenin transcriptional activity, showed decreased *β*‐catenin activity in the UB in vivo. Reduced *PRR* gene dosage in heterozygous *Six2* ^PRR^ ^+/−^ mice was associated with decreased glomerular number, segmental thickening of the glomerular basement membrane with focal podocyte foot process effacement, development of hypertension and increased soluble PRR (sPRR) levels in the urine at 2 months of age. Together, these data demonstrate that NPC PRR performs essential functions during nephrogenesis *via* control of hierarchy of genes that regulate critical cellular processes. Both reduced nephron endowment and augmented urine sPRR likely contribute to programming of hypertension in *Six2* ^PRR^ ^+/−^ mice.

## Introduction

Mammalian kidney development is driven by reciprocal inductive interactions between self‐renewing Six2^+^;Cited1^+^ nephron progenitor cells (NPCs) of the cap mesenchyme (CM) and progenitor cells in the adjacent tips of the ureteric bud (UB) (Kobayashi et al. 2008). NPCs give rise to all segments of the mature nephron from the glomerulus to the connecting tubule (Little and McMahon 2012). NPCs have a limited lifespan and are depleted around 36 weeks of gestation in humans and postnatal day 3 in mice, leading to cessation of nephrogenesis (Hinchliffe et al. 1991). Premature cessation of nephrogenesis results in reduced nephron number and is associated with renal hypoplasia, proteinuria, susceptibility to subsequent hypertension and chronic kidney disease (Brenner et al. 1988; Barker et al. 1989; Bertram et al. 2011). Thus, understanding the mechanisms that determine final nephron number might facilitate prevention of kidney and associated cardiovascular disease.

The prorenin receptor (PRR) is a receptor for prorenin and renin encoded by the *ATP6AP2* (*ATPase‐associated protein2)* gene (subsequently referred to as *PRR*) located on the X chromosome in humans (Nguyen et al. 2002). PRR is also an accessory protein of the vacuolar proton pump V‐ATPase (Ludwig et al. 1998). Global *PRR* knockout is lethal in mice, indicating an essential role of the PRR in embryonic development (Sihn et al. 2013; Song et al. 2016). In humans, *PRR* mutations are associated with a high blood pressure, left ventricular hypertrophy, and X‐linked mental retardation (Ramser et al. 2005; Hirose et al. 2009, 2011; Reidy and Rosenblum 2009). Previously, we demonstrated that nephron progenitor PRR is critical for normal kidney development and function. *PRR* ablation in Six2^+^ NPCs of the CM results in a marked decrease in the number of developing nephrons at birth and early postnatal death (Song et al. 2016). However, the transcriptome downstream of the nephron progenitor PRR and the role of PRR in programming of blood pressure have not been previously defined.

In this study, we: (1) Identified genes that are regulated by PRR in Six2^+^ NPCs using a whole‐genome expression analysis of RNA in Six2^+^cells FACS‐isolated from *Six2* ^PRR−/−^ (*Mut*) mice on embryonic day E15.5 and conducted gene ontology analysis to identify functional groups of differentially expressed genes; (2) Tested the hypothesis that reduced *PRR* gene dosage in heterozygous *Six2* ^PRR+/−^ mice (*Het*) is associated with development of hypertension during later life; and (3) Tested the hypothesis that soluble PRR (sPRR), PRR cleavage product generated subcellularly and secreted into the plasma and urine, can contribute to BP programming in *Het* mice. Our data demonstrate that NPC PRR performs essential functions during nephrogenesis *via* control of hierarchy of genes that regulate critical cellular processes. Both reduced nephron endowment and augmented urine sPRR likely contribute to programming of hypertension in *Het* mice.

## Materials and Methods

### Conditional deletion of *PRR* from nephron progenitors

*PRR*‐floxed mice were provided by Dr. Atsuhiro Ichihara (Keio University, Tokyo, Japan) (Oshima et al. 2011). To delete *PRR* conditionally in the CM and its epithelial derivatives, we used the *Six2GFPCre TGC* transgenic mice, which drives Cre expression in nephron progenitors (Kobayashi et al. 2008), and a floxed allele of the *PRR*. The resulting *Six2* ^Cre+^/*PRR* ^flox/flox^ mice represent nephron progenitor‐specific *PRR*‐knockout mice (*Mut*) (Terada et al. 2017). Control mice consisted of *Six2* ^Cre+^/*PRR* ^+/+^ littermates (*WT*). Mice were housed at the animal care facility at Tulane University at 25°C with a 12 h light/dark cycle. Animals were fed a commercial diet (Double M feed Garden & Pet, lab rodent diet 5053, ) and tap water. All experiments involving mice were approved by Tulane Institutional Animal Care and Use Committee.

### Fluorescence‐Activated Cell Sorting

**Figure 1:**
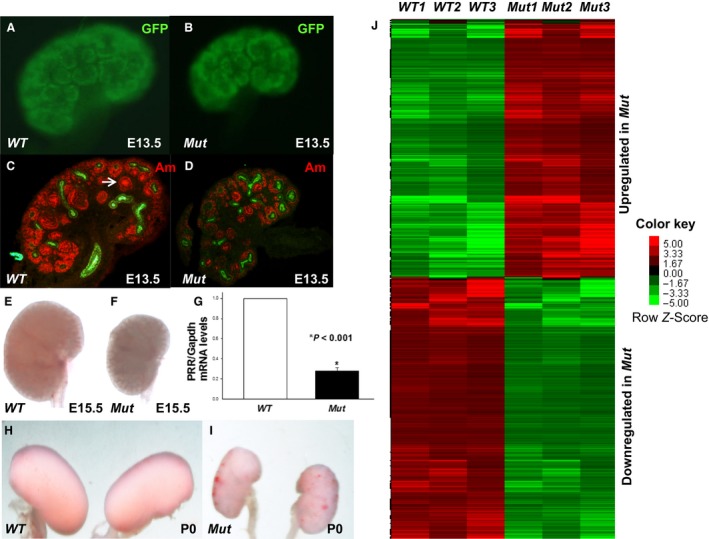
Mutant (*Mut*) kidneys are smaller than control (*WT*) kidneys and demonstrate abnormal gene regulation. (A,B): GFP immunofluorescence in E13.5 WT (A) and Mut (B) kidneys. C,D: Immunofluorescence for nephron differentiation marker amphyphisin (Am, red) on E13.5. In *WT* kidney, amphiphysin (red) staining is found in the renal vesicles (white arrow) and throughout the nephron progenitors in the nephrogenic zone (C). *Mut* kidney is smaller, has reduced amphiphysin staining and decreased formation of renal vesicles (D). Pancytokeratin (green) labels ureteric bud. (E,F) Gross images of E15.5 *Mut* and *WT* kidneys selected for microarray analysis show that *Mut* kidney (F) is smaller. (G) Bar graph shows reduced *PRR* mRNA levels in Six2^+^ cells FACS‐isolated from E15.5 *Mut* compared to *WT* kidneys. (H, I) Gross images of P0 *Mut* and *WT* kidneys show that *Mut* kidneys (*I*) are smaller in size. (J) Gene expression differs between Six2^+^ cells FACS‐isolated from *WT* and *Mut* kidneys on E15.5. Expression values of differentially regulated genes (*P* < 0.05) are shown in this heatmap, with red indicating higher expression and green lower expression. FACS, fluorescence‐activated cell sorting.

E15.5 kidneys from *Mut* and *WT* mice which express GFP in Cre^+^ cells were digested in collagenase A (25 mg/10 mL PBS) and pancreatin (100 mg/10 mL PBS) at 37°C for 15 min, dissociated by repetitive pipetting and resuspended in PBS containing 2% FBS and 10 mmol/L EDTA. The resuspended cells were filtered through 40 *μ*m nylon cell membrane (BD Falcon) and kept on ice until Fluorescence‐Activated Cell Sorting (FACS). The GFP^+^ cells were isolated using FACS Vantage and data were subsequently analyzed with Diva software v.5.02 (Becton Dickinson). RNA was isolated using Absolutely RNA Nanoprep kit (Stratagene). qRT‐PCR was performed to validate elimination of *PRR* in FACS‐isolated *Six2* ^Cre+^ cells and revealed a 72% decrease in PRR mRNA levels in Six2^+^ NPCs FACS‐isolated from *Mut* compared with *WT* kidneys (0.28 ± 0.001 vs. 1.0 ± 0, *P *< 0.001) (Fig. 1).

### Microarray and gene ontology analysis

*Six2* ^Cre+^ cells FACS‐isolated from mutant and control kidneys were divided into two random pools (mutant: *n* = 3; control: *n* = 3 pools) consisting of 10 kidneys each. Isolated RNA was hybridized to Agilent mouse GE4X44K gene expression microarray. Hybridization, scanning, and analysis were done by a core facility of the Tulane Cancer Center. Microarray data of biological triplicates of *WT* and *Mut* FACS‐isolated cells that pass QC parameters were normalized and analyzed using GeneSpring GX (Agilent, US) 12.0 software. The Benjamini‐Hochberg correction for multiple testing was applied to the set of *P* values generated for the probe coefficients, and probes with adjusted *P* < 0.05 and fold change values of >1.5 and <1.5 were determined to be statistically significant. Molecular pathway analyses were performed by Ingenuity Pathway Analysis (IPA) version 7.1 (Redwood City, CA). Microarray data are available at GEO under accession number GSE101460.

### Quantitative real‐time reverse‐transcription polymerase chain reaction (RT‐PCR)

SYBR Green quantitative real‐time RT‐PCR was conducted using MxPro QPCR software (Stratagene). The quantity of each target mRNA expression was normalized by that of GAPDH mRNA expression. RNA samples from each pool were analyzed in triplicates. PCR reaction was performed twice.

### Blood pressure measurement

Conscious tail‐cuff mean (MAP), systolic and diastolic arterial blood pressure was measured in *Six2* ^PRR+/−^ (*Het*,* n* = 3) and control *Six2* ^PRR+/+^ (*WT*,* n* = 4) mice at 2 months of age (P60) using a Visitech BP2000 system (Visitech Systems, Apex, NC). After 3 days of animal conditioning training, three consecutive cycles (10 recordings/mouse/cycle) of blood pressure readings were obtained on same day. Mean blood pressure values were calculated per each animal and used for statistical analysis.

### Measurement of plasma and urine soluble PRR (sPRR)

Urine and plasma sPRR levels were measured on P60 by enzyme‐linked immunosorbent assay (ELISA) kit according to the manufactures’ instruction (IBL American, # JP27782).

### Electron microscopy, histopathology, and immunohistochemistry

P60 *Het* and *WT* kidney tissues stored in 3% glutaraldehyde were processed and embedded by the Department of Pathology, Tulane University. Ultimately, 60 nm sections were cut and imaged using a Hitachi H‐7100 electron microscope. Glomerular number was counted in Het and WT kidneys on P60 from 3 consecutive H&E‐stained sections/kidney adjacent to the longitudinal midplane (*n* = 3 kidneys/genotype). E13.5‐E15.5 4‐*μ*m kidney sections from *Mut* and *WT* mice were processed for immunofluorescence using anti‐amphiphysin (ProteinTech, 1:200), anti‐active *β*‐catenin (ABC, Millipore, 1:400, anti‐Lotus Tetragonolobus Lectin (LTL) (1:400, Vector Laboratories), and anti‐cytokeratin (1:200, Sigma) antibodies. Immunostaining was performed by the immunoperoxidase technique with Vectastain Elite kit (Vector Laboratories, Burlingame, CA). Secondary antibodies were detected with Alexa Fluor dyes (Invitrogen). Specificity of immunostaining was documented by the omission of the primary antibody.

### *β*‐catenin‐dependent transcriptional activation in *BatGa*l^+^ mice

*Mut* or *WT* mice were crossed with *BatGal* ^+^ mice. *BatGal* ^+^ transgenic mice are a reporter strain which drives expression of nuclear *β*‐galactosidase (BatGal) under the control of multimerized LEF/TCF‐binding sites (Maretto et al. 2003). For *β*‐gal staining of whole kidneys, E13.5 kidneys (*n* = 3 kidneys/genotype) were fixed for 60 min in 0.2% Glutaraldehyde, 5 mmol/L EGTA, and 2 mmol/L MgCl_2_ in 0.1 mol/L phosphate buffer, pH 7.5 on ice, rinsed for 15 min in 0.02% Igepal, 0.01% Sodium Deoxycholate, and 2 mM MgCl_2_ in 0.1 mol/L phosphate buffer, pH 7.5, and stained by immersion in 1 mg/mL *β*‐gal solution at 37°C in the dark. Reaction was stopped by washing the kidneys with PBS. 14 *μ*m midplane frozen sections of E14.5 kidneys (*n* = 3 kidneys/genotype) were stained for *β*‐gal (1 mg/mL) at 37°C for 2 h in the dark, fixed for 30 min with 4% PFA, rinsed with water and counterstained with Eosin for 10 min. The intensity of kidney whole mount or kidney section *β*‐gal staining was assessed visually in a blinded fashion. The number of *β*‐gal positive cells per cortical area of kidney section (*n* = 3 sections per kidney/genotype) was counted.

### In situ hybridization

Section In situ hybridization (ISH) was performed on E13.5 *Mut* and *WT* kidneys as previously described (Song et al. 2016). Two embryonic kidneys per group were examined.

### Statistics

Statistical analyses were carried out upon all biologic replicates with Student's *t* test or a one‐way ANOVA, followed by Bonferroni test. Data are presented as Mean ± SEM. A *P* < 0.05 was considered statistically significant.

## Results

### Effect of PRR deficiency in Six2^+^ nephron progenitors on Six2^+^ cell transcriptome

**Figure 2:**
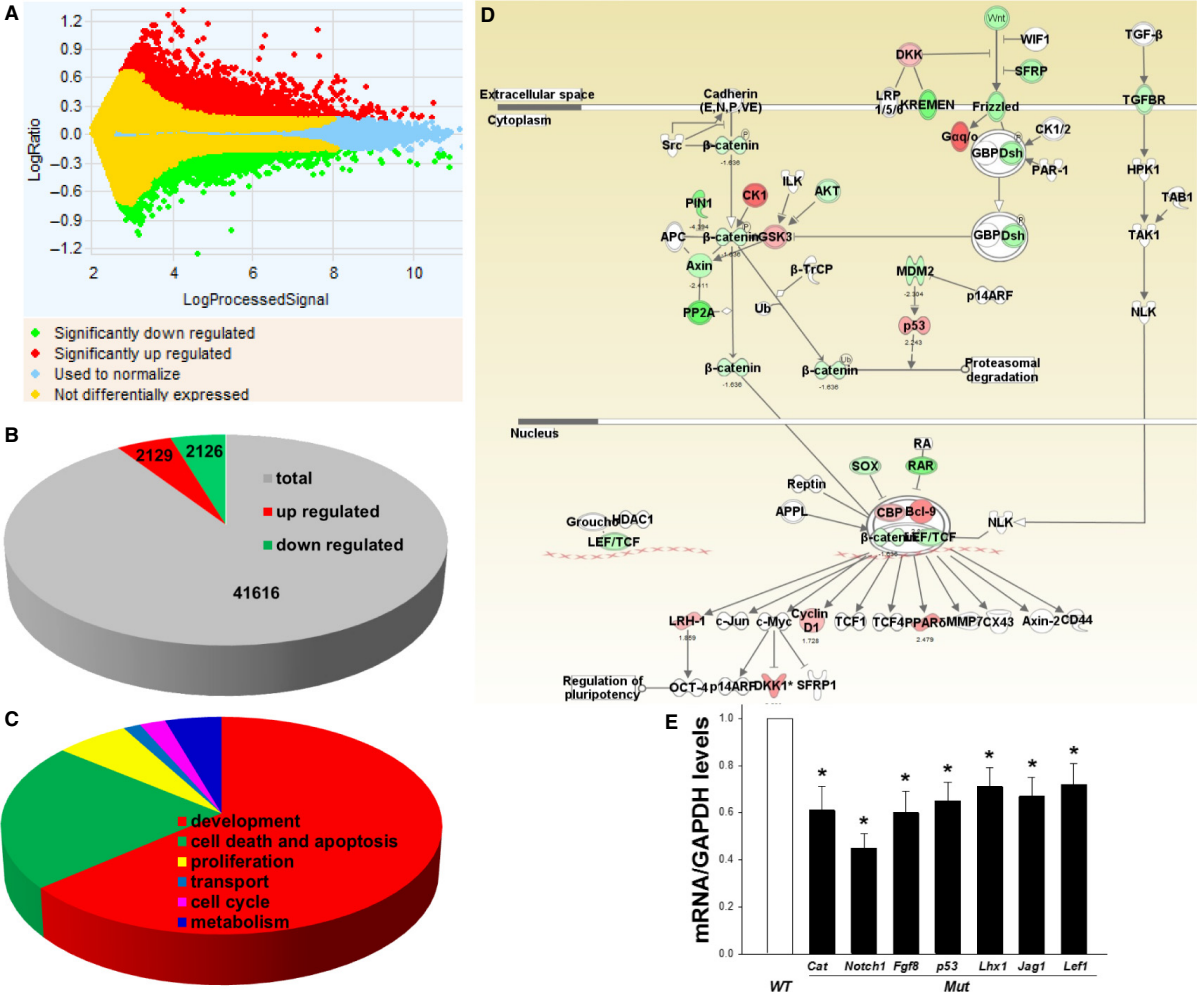
(A) Microarray analysis of gene expression in Six2^+^ cells FACS‐isolated from mutant (*Mut*) kidneys on E15.5 (A). (B–D): Ingenuity Pathway Analysis (IPA) and representation of the Wnt‐*β*‐catenin signaling pathway. (D) Red/pink: indicates upregulated genes, green: indicates downregulated genes. (E) Validation of microarray data by real‐time RT‐PCR comfirmed a significant downregulation of *β*‐*catenin* (Cat), *Notch1, Fgf8, p53, Lhx1, Jag1, and Lef1* mRNA expression in NPCs FACS‐isolated from E15.5 *Mut* compared with *WT* mice. *P < 0.01 versus other groups. FACS, Fluorescence‐activated cell sorting.

Previously, we demonstrated that mice with conditional deletion of *PRR* in the Six2^+^ nephron progenitors have a marked decrease in the number of developing nephrons and small cystic kidneys at birth, and early postnatal death (Song et al. 2016). Here, we investigated PRR‐directed transcriptome using whole‐genome‐based analysis of gene expression in Six2^+^ nephron progenitors FACS‐isolated from *Mut* and *WT* kidneys on E15.5. On E13.5, *Mut* kidneys are smaller than *WT* kidneys and demonstrate reduced expression of nephron differentiation marker amphiphysin (Fig. 1). On E15.5, *Mut* kidneys remain smaller than controls and demonstrate abnormal gene regulation in NPCs (Fig. 1). Analysis of the microarray data by hierarchical cluster analysis revealed a low level of variability among biological replicates (Fig. 1). Of the ~44,000 transcripts represented on the GE4X44K array, data analysis identified 4255 (10.2%) differentially expressed genes [2129 (5.1%) upregulated, 2126 (5.1%) downregulated] (>1.5‐fold change, *P* < 0.05) (Fig. 2). To determine changes in expression levels of individual genes, we identified the top 10 up‐ and downregulated genes (Tables 1, 2). The magnitude of changes in top 10 gene expression was 9–35‐fold for downregulated genes and 5–8‐fold in upregulated genes.

**Table 1:** Top 10 downregulated genes in Six2^+^ cells FACS‐isolated from Six2^PRR−/−^ kidneys

Gene name	Fold change	GO biological process	GO molecular function
Hoxb8	−35.6	Dorsal spinal cord development; cytokine‐mediated signaling pathway; multicellular organismal development; positive regulation of JAK‐STAT; anterior/posterior pattern specification; positive regulation of cell proliferation	DNA binding; cytokine activity; growth factor activity; sequence‐specific DNA binding
Mpst	−18.5	Hydrogen sulfide biosynthetic process	Transferase activity; thiosulfate sulfurtransferase activity; 3‐mercaptopyruvate sulfurtransferase activity
Cmtm4	−12.2	Chemotaxis	Cytokine activity
Mdfi	−11.8	Activation of JUN kinase activity; dorsal/ventral axis specification; negative regulation of DNA binding; regulation of Wnt signaling pathway	Protein binding; transcription factor binding
Aph1a	−11.4	Proteolysis; protein processing; metanephros development; Notch signaling pathway; Notch receptor processing; integral component of plasma membrane	Protein binding; peptidase activity; endopeptidase activity
Mdfi	−9.9	Activation of JUN kinase activity; dorsal/ventral axis specification; negative regulation of DNA binding; regulation of Wnt signaling pathway	Transcription factor binding; protein binding
Atg4b	−9.68753	Transport; autophagy; proteolysis; protein transport; autophagosome assembly; positive regulation of autophagy; positive regulation of protein catabolic process	Protein binding; peptidase activity; hydrolase activity; endopeptidase activity; cysteine‐type peptidase activity; cysteine‐type endopeptidase activity
Atp1a1	−8.80377	Transport; ion transport; cation transport; dephosphorylation	ATP binding; ADP binding; protein binding; ATPase activity; ankyrin binding; metal ion binding
Stk32a	−8.79321	Phosphorylation; protein phosphorylation	ATP binding; kinase activity; metal ion binding; nucleotide binding; transferase activity; protein kinase activity; protein serine/threonine kinase activity; transferase activity, transferring phosphorus‐containing groups
Gpc2	−8.67856	Smoothened signaling pathway; neuron differentiation	Heparan sulfate proteoglycan binding

**Table 2:** Top 10 upregulated genes in Six2+ cells FACS‐isolated from Six2^PRR−/−^ kidneys

Gene name	Fold change	GO biological process	GO molecular function
Ahdc1	7.6	Any process specifically pertinent to the functioning of integrated living units	Elemental activities, such as catalysis or binding
Ebf1	6.2	Multicellular organismal development regulation of transcription, positive regulation of transcription	DNA binding protein dimerization activity, C2H2 zinc finger domain binding protein binding, metal ion binding
Tmem74	6.2	Autophagy	Elemental activities, such as catalysis or binding
2010106E10Rik	5.7	Any process specifically pertinent to the functioning of integrated living units	Elemental activities, such as catalysis or binding, describing the actions of a gene product at the molecular level.
Cd247	5.5	Cell surface receptor signaling pathway	Protein binding; transmembrane signaling receptor activity
Hbb‐y	5.5	Transport; protein heterooligomerization; negative regulation of transcription from RNA polymerase II promoter	Hemoglobin alpha binding; oxygen transporter activity; heme binding; metal ion binding; oxygen binding; iron ion binding
Cma1	5.5	Proteolysis; positive regulation of angiogenesis; interleukin‐1 beta biosynthetic process	Peptide binding; catalytic activity; peptidase activity; hydrolase activity
Kirrel3	5.4	Glomerulus morphogenesis; neuron projection morphogenesis; hemopoiesis; neuron migration	Protein binding; PDZ domain binding
4930592I03Rik	5.4	Any process specifically pertinent to the functioning of integrated living units	Elemental activities, such as catalysis or binding
Il5	5.4	Immune response; cytokine‐mediated signaling pathway positive regulation of JAK‐STAT cascade; positive regulation of cell proliferation	Growth factor activity; cytokine activity interleukin‐5 receptor binding

### Gene ontology analysis suggests abnormal regulation of kidney development and function genes

Next, we performed a gene ontology analysis to identify functional groups of differentially expressed genes by Ingenuity Pathway Analysis (IPA). The major GO biological processes and molecular functions enriched within top downregulated genes included cancer, cellular assembly and organization, cellular function, development, morphology, cell death and survival (Table 3). These categories are highly consistent with the mutant phenotype and a putative role of PRR in controlling the expression of genes involved in nephrogenesis and kidney development (Song et al. 2016). We next investigated the list of up‐ and downregulated genes for genes which redundant/deficient state is associated with aberrant nephrogenesis. We identified seven downregulated genes that met these criteria (Table 4). The expression of PRR‐dependent genes in NPCs was validated by qRT‐PCR (Fig. 2). Consistent with predominant morphogenetic events that occur at E15.5, such as nephron formation (nephrogenesis) and differentiation (establishment of specialized cell types marking specific nephron segments), we observed enrichment of IPA terms such as cellular assembly and organization, cell morphology, development, cell death and survival within the downregulated gene set (Table 3). To identify genes that may function during early nephrogenesis, we crossreferenced all downregulated genes found in this study with the microarray expression data in the CM lineage available in GUDMAP (Harding et al. 2011). A total of 238 genes were referenced in GUDMAP as expressed in the E15.5 CM lineage (Harding et al. 2011). Of 2126 genes downregulated in our array, 12 were referenced as expressed in the CM lineage in GUDMAP. Table 5 shows top 10 of these 12 downregulated genes in our array. The majority of downregulated genes belonged to kidney/embryonic development, cell morphogenesis, regulation of transcription, cytoskeleton organization, cell cycle, cell proliferation categories, consistent with the role for the NPC PRR in nephrogenesis.

**Table 3:** Top diseases and biological functions altered in Six2^+^ cells FACS‐isolated from Six2^PRR−/−^ kidneys

Category	P‐value	Number of molecules
Diseases and disorder		
Organismal injury and abnormalities	1.27E‐04‐2.66E‐34	2415
Gastrointestinal disease	1.27E‐04‐2.66E‐34	2424
Hepatic system disease	1.28E‐04‐2.49E‐30	1782
Reproductive system disease	1.06E‐04‐2.57E‐18	1076
Cancer	7.04E‐05‐3.88E‐17	964
Molecular and cellular functions		
Cellular assembly and organization	1.11E‐04‐2.15E‐23	552
Cellular function and maintenance	7.14E‐05‐2.15E‐23	709
Cell morphology	1.21E‐04‐2.74E‐21	720
Cellular development	1.02E‐04‐4.60E‐16	823
Cell death and survival	1.27E‐04‐8.11E‐16	858
Physiological System Development and Function		
Organismal survival	1.50E‐05‐6.52E‐29	654
Embryonic development	1.21E‐04‐1.22E‐20	691
Organismal development	1.21E‐04‐1.22E‐20	939
Nervous system development and function	1.21E‐04‐2.25E‐17	541
Tissue morphology	1.21E‐04‐3.34E‐17	704
Nephrotoxicity		
Renal necrosis/cell	1.00E00‐7.18E‐06	105
Death nephrosis renal	5.86E‐01‐6.33E‐03	16
Inflammation renal nephritis renal	1.00E00‐1.85E‐02	52
Regeneration	1.00E00‐1.85E‐02	52

**Table 4:** Ingenuity pathway analysis: selected functional categories of genes upregulated and downregulated in FACS‐isolated Six2^+^ cells from Mut (Six2 ^PRR−/−^) mice on E15.5

Category	P Value	Genes in test set
Formation of embryonic tissue		
Upregulated	1.07E‐03	AXIN1, DVL1, HSP90B1, SMAD3, SOX17, VEGFA
Downregulated	1.07E‐03	CITED2, CTNNB1, FGF1, FGF8, FGFR2, HNF1B, LEF1, LHX1, NCAM1, NOTCH1, HES
Abnormal morphology		
Upregulated	1.09E‐04	ATP13A2, MDM2, SMAD3, SMAD5
Downregulated	1.09E‐04	ATP1B2, ATP2C1, BAD, CCND1, CITED2, CYP1A2, DVL1, IL2,LEF1, LTA, NCAM1, PBX1, PDGFRB, P53
Formation of kidney		
Upregulated	4.90E‐05	BMP6, SMAD3
Downregulated	4.90E‐05	CITED2, CTNNB1, FGF8, FGFR1, FGFR2, JAG1, LHX1, PBX1, PDGFRB, SPRY1, TCF21, P53
Cell death and survival		
Upregulated	1.16E‐05	BMPR1A, JAG2, SMAD5, SOX17
Downregulated	1.16E‐05	ATP2C1, BCL2, CTNNB1, EYA1, GATA6, IL10, MAP3K1, P53

**Table 5:** Top 10 downregulated genes crossreferenced with the microarray expression data available in GUDMAP

Symbol	Description	FC	E15.5 CM	Go Biological Process	Go Molecular Function
Pclo	Piccolo Presynaptic Cytomatrix Protein	−3.20	20.58	GO:0007010cytoskeleton organiza‐tion; GO:0007416 synapse assembly	GO:0005509 calcium ion binding; GO:0005522, profilin binding
Cdc14a	Cell Division Cycle 14A	−2.31	5.71	GO:0006470 protein dephos‐phorylation; GO:0007049 cell cycle; GO:0008283 cell proliferation	GO:0004721 phosphoprotein phosphatase activity; GO:0004722 protein serine/threonine phosphatase activity
Adcy8	Adenylate Cyclase 8	−2.08	17.53	GO: 0003091 renal water homeostasis; GO:0006171 cAMP biosyn‐thetic process	GO:0004016 adeny‐late cyclase activity; GO:0005524 ATP binding
Slc1a3	Solute Carrier Family 1 Member 3	−2.08	10.81	GO:0003333amino acid transmem‐brane transport GO:0006536 glutamate metabolic process	GO:0005313 L‐glutamate transmembrane transporter activity; GO:0015171 amino acid transmembrane transporter activity
Has2	Hyaluronan Synthase 2	−1.95	5.44	GO:0001822 kidney development	GO:0016740 transferase activity
Trim16	Tripartite Motif Containing 16	−1.86	1.20	GO:0006355 regulation of transcrip‐tion, DNA‐templated; GO:0043966 histone H3 acetylation	GO:0003677 DNA binding; GO:0005515 Protein binding
Ednra	Endothelin Receptor Type A	−1.77	2.80	GO: 0001569 branching involved in blood vessel morphoge‐nesis; GO:0001701 in utero embry‐onic development	GO:0004871signal transducer activity; GO:0004930 G‐protein coupled receptor activity
Chst9	Carbohydrate Sulfotransferase 9	−1.74	11.08	GO:0005975 carbohydrate metabo‐lic process; GO:0006790 sulfur compound metabolic process	GO:0008146sulfotransferase activity; GO:0016740transferase activity
Bcl2	BCL2, Apoptosis Regulato	−1.66	7.69	GO:0000902 cell morphogenesis;GO:0000209 protein polyubiquitination	GO:0002020 protease binding; GO:0005515 Protein binding
Enc1	Ectodermal‐Neural Cortex 1	−1.58	3.75	GO:0007275 multicellular organism development; GO:0007399 nervous system development	GO:0005515 Protein binding

FC, fold change; CM, cap mesenchyme.

### NPC PRR promotes nephrogenesis through canonical Wnt/*β*‐catenin signaling

**Figure 3:**
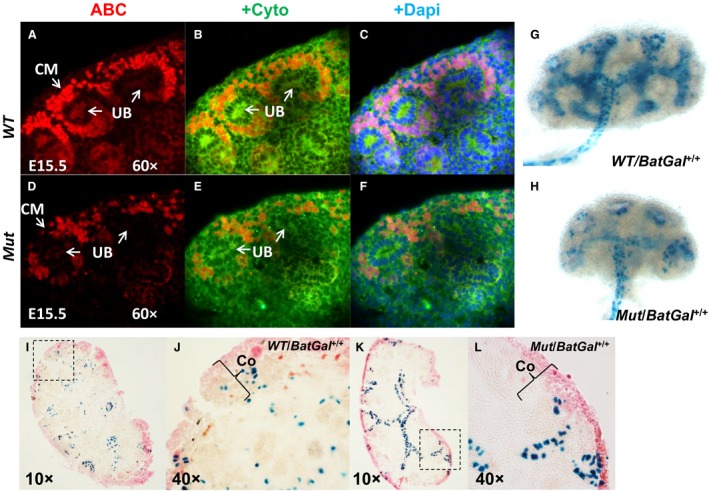
Effect of conditional *PRR* deletion in Six2^+^ NPCs on active *β*‐catenin (ABC) protein levels, *β*‐gal staining, and *Gdnf* mRNA expression in the kidney. (A–F): ABC immunoflurescence shows apparent reduction in ABC levels (red staining) in the CM of mutant (*Mut*,* D*‐*F*) compared to control (*WT*, A–C) E14.5 kidneys. Dapi‐ blue, anti‐pancytokeratin (Cyto)‐ green staining. *G*,* H*: Whole mount *β*‐gal staining (blue) of E13.5 kidneys isolated from *Mut*/*BatGal* ^+/+^ and *WT*/*BatGal* ^+/+^ mice. Representative images show that *Mut*/*BatGal* ^+/+^ kidney is smaller and shows weaker Bat/Gal activity in the UB compared to control *WT*/*BatGal* ^+/+^ kidney. (I–L): *β*‐gal staining (blue) of E14.5 kidney sections shows apparent reduction in staining in the cortex (Co) of *Mut*/*BatGal* ^+/+^ compared with *WT*/*BatGal* ^+/+^ mice. J, L: High power images of areas shown by dashed line insets in I and K.

**Figure 4:**
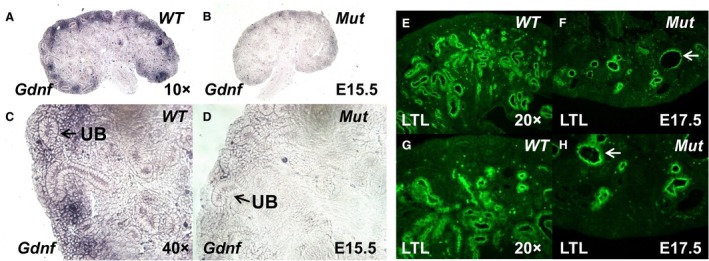
(A–D) Section in situ hybridization of E14.5 kidneys shows apparent decrease in *Gdnf* mRNA expression in the mesenchyme of mutants (*Mut*) compared to controls (*WT*, dark blue staining). (E–H): Kidney sections of E17.5 *Mut* and *WT* mice stained with anti‐Lotus Tetragonolobus Lectin (LTL, green) antibody show paucity and dilation (*N*,*P*, arrows) of tubules of proximal tubule origin in *Mut* kidneys.

Previously, we demonstrated that canonical Wnt/*β*‐catenin signaling is required downstream of NPC PRR for mesenchyme to epithelial transition (MET) in vitro (Song et al. 2016). IPA analysis of current microarray data revealed downregulation of the Wnt/*β*‐catenin pathway in FACS‐isolated mutant Six2^+^ NPCs, consistent with critical role of intact Wnt/*β*‐catenin signaling downstream of NPC PRR to direct nephrogenesis (Fig. 2) (Park et al. 2007; Marose et al. 2008; Song et al. 2016). In accord with IPA findings, expression of the functionally active form of *β*‐catenin protein (ABC) dephosphorylated on ^Ser^37 or ^Thr^41 was reduced in the CM of E15.5 *Mut* kidneys (Fig. 3). In addition, ABC immunofluorescence was decreased in the UB of *Mut* compared to *WT* kidneys (Fig. 3). To provide genetic proof that PRR signaling induces *β*‐catenin transcriptional activity in vivo in NPCs, we tested the hypothesis that metanephroi of double‐transgenic *Mut*/*BatGal* ^+^ mice exhibit decreased *β*‐catenin activity in the CM. Whole mount LacZ staining on E13.5 was apparent only in the UB in both *Mut*/*BatGal* ^+^ and *WT*/*BatGal* ^+^ mice and was reduced in the UB of *Mut*/*BatGal* ^+^ mice (Fig. 3). Given that targeted inactivation of *β*‐catenin in the UB cell lineage in mice causes reduced UB branching, (Bridgewater et al. 2008) a UB phenotype similar to what we observed in *Mut* mice,(Song et al. 2016) our findings of reduced *β*‐catenin activity in the UB of *Mut* mice demonstrate an additional pathological hit in which decreased *β*‐catenin in NPCs of *Mut* kidneys leads to reduced *β*‐catenin activity in the ureteric epithelium. Thus, reduced UB branching observed in *Mut* mice (Song et al. 2016) may result from both decreased nephrogenesis and reduced levels of transcriptionally active *β*‐catenin within the ureteric epithelium. Because overexpression of *β*‐catenin in the metanephric mesenchyme was reported to induce *β*‐catenin activity in the UB *via* upregulation of glial cell‐derived neurotrophic factor (Gdnf), secreted signaling molecule expressed throughout the CM and necessary for kidney development, (Sarin et al. 2014) we tested whether reduced *β*‐catenin activity in the UB of *Mut* kidneys is due to decrease in Gdnf expression in the mesenchyme. ISH showed apparent reduction in *Gdnf* mRNA expression in the mesenchyme of *Mut* compared with *WT* kidney (Fig. 4). Inability to detect LacZ staining in the CM using whole mount kidney may result from reduced ability to visualize staining due to high tissue thickness or density, or to relatively lower levels of *β*‐catenin activity in the CM versus the UB epithelia. To determine the effect of PRR signaling on *β*‐catenin transcriptional activity in NPCs *in vivo* with greater precision, we examined LacZ staining in *WT* and *Mut* E14.5 kidney sections. The number of X‐gal‐positive cells in the cortex, where active nephrogenesis occurs, was reduced in *Mut* compared to *WT* kidneys (3.1 ± 0.7 vs. 15.2 ± 1.6, *P* < 0.01) (Fig. 3). These findings are consistent with decreased *β*‐catenin activity in the CM of *Mut*/*BatGal* ^+^ mice. Mechanistically, decreased *β*‐catenin activity in NPCs in *PRR Mut* mice may reduce UB *β*‐catenin signaling in a paracrine‐dependent manner through downregulation of Gdnf levels in the CM.

### Novel PRR‐dependent genes and control of nephrogenesis

Notch signaling in NPCs promotes initiation of nephrogenesis and the formation of proximal tubules (Cheng et al. 2007; Chung et al. 2016). Previously, we demonstrated reduced expression of Jagged1 (Jag1), the major Notch ligand in the process of nephrogenesis, in *PRR Mut* kidneys (Song et al. 2016). Since our array identified downregulation of *Notch1* and of Notch‐target gene *Hes5* in *Mut* NPCs, we investigated the formation of proximal tubules in *Mut* kidneys. In *WT* kidneys, NPCs formed LTL‐positive proximal tubules (Fig. 4). In contrast, *Mut* kidneys had paucity and marked dilation of LTL‐positive proximal tubules. Proximal tubular defects observed in *Mut* mice are most likely due to premature exhaustion of NPCs (Song et al. 2016). Our findings of reduced *Notch1* and *Hes5* expression in *Mut* NPCs suggest that additional mechanism may involve aberrant Notch signaling downstream of NPC PRR in a subset of NPCs that were able to differentiation into the proximal tubule segment of the nephron. Expression of *Jag1*, the major Notch ligand in the process of nephrogenesis and a marker for renal vesicle (RV) nephron stage, was also reduced in *Mut* NPCs (Fig. 1, Table 4). p53, a tumor suppressor that regulates cell‐cycle, differentiation, and apoptosis pathways, is also important for normal embryonic kidney development. Global *p53* deletion in mice results in UB ectopia, reduced UB branching, and hypoplastic metanephroi (Saifudeen et al. 2009). Reduced p53 mRNA levels in *Mut* NPCs suggest that p53 functions downstream of the NPC PRR to promote nephrogenesis.

### Reduced *PRR* gene dosage in nephron progenitors results in development of hypertension during later life

**Figure 5:**
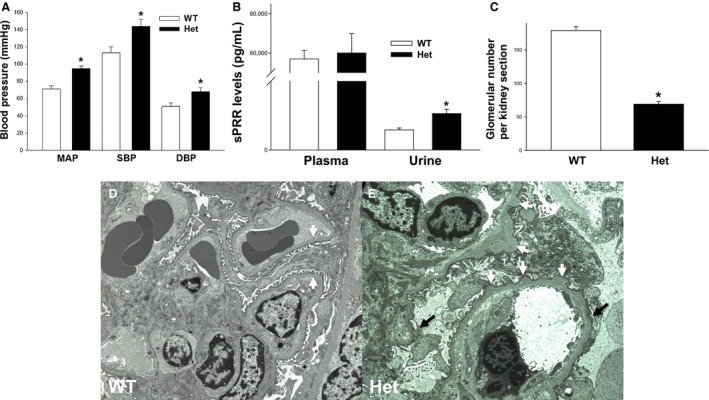
Reduced *PRR* gene dosage in Six2^+^ NPCs results in decreased glomerular number, altered glomerular basement membranes (GBM) ultrastructure, elevated blood pressure and increased soluble PRR (sPRR) levels in the urine at 2 months of age. (A): *Het* mice have significantly increased mean (MAP), systolic (SBP), and diastolic (DBP) blood pressure at 2 months of age. *P < 0.01. (B) *Het* mice have significantly increased sPRR levels in the urine. *P < 0.05. (C) Glomerular number is significantly decreased in *Het* mice. (D, E) Electron micrograph of *WT* kidney section (D) shows normally appearing GBM with intact podocyte foot processes (white arrows). *Het* kidney section (E) shows focal podocyte foot process effacement (black arrows) with focal microvillus change and irregularities in the GBM with segmental thickening (white arrows).

We previously demonstrated that *Mut* (*Six2* ^PRR−/−^) mice exhibit reduced nephron number at birth due to precocious depletion of NPC pool (Song et al. 2016). Low nephron number has been recognized as a determinant of susceptibility to hypertension both in animal models (Woods et al. 2004) and in humans (Brenner et al. 1988; Hughson et al. 2006). In humans, nephrogenesis is completed by ~36 weeks of gestation and in rodents‐ by ~3 days after birth (Hinchliffe et al. 1991; Reidy and Rosenblum 2009). Because *Mut* mice did not survive beyond the first 48 h of life, we evaluated the effect of reduced *PRR* gene dosage in *Six2* ^PRR+/−^ (*Het*) mice on nephron number, glomerular basement membrane (GBM) ultrastructure, and blood pressure at 2 months of age. While the number of glomeruli per kidney section was reduced in *Het* compared with control mice (69 ± 4.0 vs. 178 ± 4.9, *P* < 0.001), conscious tail‐cuff mean (95.5 ± 2.8 vs. 70.4 ± 3.8, p < 0.01), systolic (143 ± 5.3 vs. 113 ± 6.5, *P* < 0.01), and diastolic (67 ± 4.5 vs. 51 ± 4.0, *P* < 0.05) arterial blood pressure was increased in *Het* mice (Fig. 5). Electron microscopy (EM) of P60 *Het* kidney section showed segmental GBM thickening with focal microvillus changes and focal podocyte foot process effacement, a hallmark of glomerular injury leading to proteinuria (Fig. 5). Thus, reduced *PRR* gene dosage in nephron progenitors results in decreased glomerular number, abnormal GBM, and increased blood pressure later in life.

### Reduced *PRR* gene dosage in nephron progenitors results in increased urinary soluble PRR (sPRR) levels

The full‐length PRR protein (39 kDa) is cleaved by enzyme furin to generate sPRR (28 kDa) which is secreted into the extracellular space and is ultimately found in the blood and urine (Cousin et al. 2009; Gonzalez et al. 2011). Given that elevated plasma sPRR levels correlate positively with histological evidence of renal tissue damage in humans (Ohashi et al. 2016) and that sPRR is functionally active in the urine,(Gonzalez et al. 2011) we next measured plasma and urine sPRR levels in *Het* and control mice at 2 months of age. While plasma sPRR levels did not differ (60,045 ± 9070 vs. 56,166 ± 4380 pg/mL, *P* = 0.72), urinary sPRR levels were increased in *Het* compared with control mice (263 ± 30 vs. 146 ± 14 pg/mL, *P* < 0.05) (Fig. 5). Because it is unknown whether sPRR (28 kDa) is filtered from plasma to the urine and in view of abundant PRR expression in the collecting duct (CD), we speculate that urinary sPRR excretion likely reflects cleavage of full‐length PRR in the CD and intratubular sPRR secretion.

## Discussion

**Figure 6:**
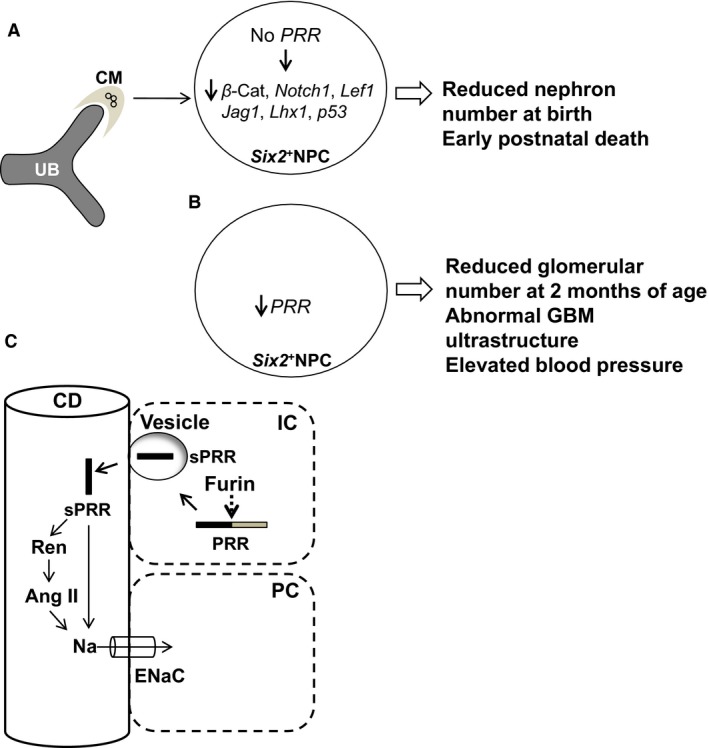
Model of nephron progenitor cell (NPC) prorenin receptor (PRR) function in nephrogenesis, glomerular basement membrane (GBM) ultrastructure, and hypertension. (A) Lack of PRR in *Six2* ^+^ NPCs results in congenital decrease in nephron number *via* reduction in the expression of NPC pathways critical for nephrogenesis. (B) Reduced PRR gene dosage in NPCs results in reduced glomerular number, abnormal GBM ultrastructure, and elevated blood pressure at 2 months of age. (C): Reduced PRR gene dosage in NPCs results in compensatory increase in soluble PRR (sPRR) levels in the urine. We propose that in intercalated cell (IC) of the collecting duct (CD), full‐length PRR is cleaved by furin to generate sPRR which is secreted in vesicle into the CD lumen. In the CD lumen, sPRR may lead to elevated blood pressure by: a) direct stimulation of ENaC channel activity and increased Na reabsorption in the principal cell (PC) of the CD; and/or b) enhancement of enzymatic activity of renin/prorenin (Ren) leading to angiotensin II (Ang II) generation and increased renal Na reabsorption.

Previously, we demonstrated that conditional deletion of *PRR* in Six2^+^ NPCs results in early neonatal death, a marked decrease in the number of developing nephrons at birth and small cystic kidneys (Song et al. 2016). Yet, the transcriptome downstream of the nephron progenitor PRR that underlies its actions has not been previously defined in vivo. In this study, we first identified genes that are regulated by PRR in Six2^+^ NPCs using a whole‐genome expression analysis of RNA in mice with *PRR* deficiency targeted to Six2^+^ NPCs (*Mut*) and conducted gene ontology analysis to identify functional groups of differentially expressed genes. The expression of seven genes, *β*‐catenin, *Notch1*,* Lef1, Lhx1, Jag1, Fgf8, and p53* with expression in NPCs for which deficient state is associated with aberrant nephrogenesis was downregulated in *PRR Mut* NPCs. Bioinformatic analyses of our data demonstrated that NPC PRR performs essential functions during nephrogenesis *via* control of hierarchy of genes that regulate embryonic and cellular development, renal regeneration, cellular assembly and organization, cell morphology, cell death and survival. Next, we demonstrated that reduced *PRR* gene dosage in NPCs in *Six2* ^PRR+/−^ (*Het*) mice is associated with reduced number of glomeruli, ultrastructural changes in the GBM, and development of hypertension at 2 months of age. We then showed that levels of soluble PRR (sPRR), PRR cleavage product generated subcellularly and secreted extracellularly, are increased in the urine of *Het* compared with control *WT* mice at 2 month of age. Our data show that: (1) NPC PRR performs essential functions during nephrogenesis *via* control of hierarchy of genes that regulate critical cellular processes; and (2) Both reduced nephron endowment and augmented urine sPRR likely contribute to programming of hypertension in *Het* mice. Our results support a model in which lack of *PRR* in NPCs results in aberrant expression of genes critical for nephrogenesis in NPC lineage (Fig. 6). Reduced *PRR* gene dosage in NPCs results in reduced glomerular number, abnormal GBM ultrastructure, and elevated blood pressure at 2 months of age. We propose that reduced *PRR* gene dosage in NPCs results in compensatory increase in sPRR levels in the CD which may lead to elevated blood pressure by stimulation of ENaC channel activity and increased Na^+^ reabsorption in the CD (Fig. 6).

### NPC *PRR*‐dependent genes and control of nephrogenesis

The balance of NPC self‐renewal and differentiation into nephrons ultimately determines nephron endowment (final nephron number) (Little and McMahon 2012). Because nephrogenesis ends by 36 weeks of gestation in humans, nephron regeneration postnatally is not possible. Reduced nephron endowment is associated with renal hypoplasia, susceptibility to subsequent hypertension and chronic kidney disease (CKD) (Brenner et al. 1988; Bertram et al. 2011). Several key signaling pathways, including Wnt/*β*‐catenin, Six2, Eya1, Notch, Osr1, Pax2, Sall1, Bmp7, Fgf2, Fgf9, and Fgf20, are required in NPCs for nephrogenesis (Self et al. 2006; Blank et al. 2009; Brown et al. 2011, 2013; Barak et al. 2012; Kanda et al. 2014; Chung et al. 2016). We observed downregulation of several of these genes for which deficient state is associated with aberrant nephrogenesis (Table 4). Wnt/*β*‐catenin is required for both the self‐renewal and differentiation of NPCs (Carroll et al. 2005; Park et al. 2007; Karner et al. 2011). Both conditional inactivation or stabilization of *β*‐catenin in the metanephric mesenchyme in mice disrupts nephrogenesis (Park et al. 2007; Sarin et al. 2014). Thus, a finely tuned level of Wnt/*β*‐catenin signaling in the mesenchyme is essential for proper nephrogenesis. Wnt/*β*‐catenin pathway activity was reduced in both NPCs and in the UB of *PRR Mut* mice, indicating a critical role for *β*‐catenin downstream of NPC PRR in metanephric kidney development. In addition, *β*‐catenin transcriptional activity in vivo was decreased in the UB of *Mut* mice. Our data suggest that decreased *β*‐catenin activity in NPCs of *Mut* mice might reduce UB *β*‐catenin signaling in a paracrine‐dependent manner through downregulation of *Gdnf* levels in the mesenchyme. Thus, reduced *β*‐catenin activity in the UB of *Mut* mice may represent an additional pathological hit that, together with decreased *β*‐catenin signaling in NPCs, contributes to reduced UB branching, decreased number of nephrons and small cystic kidneys observed at birth in *Mut* mice (Song et al. 2016). We also observed decreased expression of *Notch 1*,* p53,* and *Fgf8* in *Mut* mice. Notch signaling in NPCs is crucial for nephrogenesis through downregulation of *Six2*, a transcription factor required for NPC maintenance (Chung et al. 2016). It is possible that reduced *Notch* expression in *Mut* mice might inhibit nephron induction *via* disinhibition of Six2. Murine double minute 2 (Mdm2)‐p53 pathway is essential to the maintenance of the NPC niche (Hilliard et al. 2014). Reduced *p53* mRNA levels in *Mut* NPCs suggest that p53 functions downstream of the NPC PRR to promote nephrogenesis. Reduced *p53* expression may be due to increased levels of *Mdm2*, a p53 ubiquitin ligase, in *Mut* NPCs. Fgf8 is required for nephron development (Grieshammer et al. 2005; Perantoni et al. 2005). Mice with pan‐mesodermal loss of *Fgf8* show lack of CM formation by E16.5 and block in vesicle progression to comma‐ and S‐shaped nephrons (Perantoni et al. 2005). Because Fgf8 is one of the earliest markers that demarcate the transition of NPCs to pre‐tubular aggregates and RVs, decreased nephron number in *Mut* kidneys at birth may be due to block in MET from reduced *Fgf8* expression (Song et al. 2016).

### NPC PRR and developmental programming of hypertension

Previously, we identified NPC PRR as critical regulator of congenital nephron endowment (Song et al. 2016). Here, we demonstrate that even reduced *PRR* gene dosage in NPCs in *Het* mice is associated with reduced glomerular number, aberrant GBM ultrastructure with focal podocyte foot process effacement and increased blood pressure at 2 months of age. It is now well established that adverse events in utero can affect nephrogenesis and result in low nephron endowment (Bertram et al. 2011). Brenner et al. (1988) hypothesized that low congenital nephron number might explain why some individuals are more susceptible to hypertension and renal disease than others later in life (developmental programming). The major factors influencing in utero environment that are associated with a low final nephron number include uteroplacental insufficiency, maternal low protein diet, hyperglycemia, vitamin A deficiency, exposure to or interruption of endogenous glucocorticoids (Chong and Yosypiv 2012). Reduced nephron number may be associated with reduced filtration surface area, thus limiting sodium excretion and leading to higher blood pressure or inappropriate activation of other vasoactive systems (e.g., renin‐angiotensin system, RAS). Even though Brenner's hypothesis offers an explanation for the association of a reduced nephron number with hypertension and renal disease, no definitive proof has been found that low nephron endowment per se causes increased risk for hypertension or renal injury. For example, hypertension is not associated with glomerular number in humans (Hughson et al. 2006). What is the relevance of our study to kidney development and pathogenesis of hypertension in humans? In human neonates, PRR immunoreactivity is present in the glomeruli, proximal tubules, collecting ducts, and arteries (Terada et al. 2017). In addition, the levels of PRR protein expression in neonatal kidney correlate inversely with gestational age and are higher in premature compared with full‐term neonates (Terada et al. 2017). These findings suggest that PRR may play an important role in kidney development in humans.

### Increased urinary sPRR and developmental programming of hypertension

We demonstrate that urinary levels of sPRR are increased in hypertensive *Het* mice at 2 months of age. Elevated sPRR levels in the urine of *Het* mice may reflect kidney tissue damage, the status of the kidney tissue RAS activity or act independently of the RAS and contribute to programming of increased blood pressure in these mice. Mechanistically, increased urinary sPRR levels can enhance activity of renin/prorenin in the CD leading to generation of angiotensin II (Ang II), the major vasoactive peptide of the RAS (Gonzalez et al. 2011). In addition, sPRR may act independent of Ang II/Ang II receptors to induce expression and activity of the epithelial sodium channel (ENaC) in the CD, thus contributing to an increase in blood pressure *via* enhanced Na^+^ reabsorption in the CD (Ramkumar et al. 2016).

In summary, NPC PRR performs essential functions during nephrogenesis *via* control of hierarchy of genes that regulate critical cellular processes in NPC lineage. Reduced *PRR* gene dosage in NPCs is associated with reduced number of glomeruli, ultrastructural changes in the GBM, and development of hypertension at 2 months of age. We propose that both reduced nephron endowment and augmented urine sPRR may contribute to programming of hypertension in mice with reduced *PRR* gene dosage in NPCs. This analysis provides important clues on the molecular etiology of reduced nephron endowment that will assist in the development of novel approaches aimed at early diagnosis and counseling of patients with renal hypoplasia/hypodysplasia and identification of targets for treatment or prevention.

## Conflict of Interest

Nothing to disclose.
